# Hidden Encephalopathy: A Mysterious Case

**DOI:** 10.7759/cureus.78687

**Published:** 2025-02-07

**Authors:** Raquel Dias Moura, Mariana Estrela Santos, Francisca Carmo, João Miranda, Telmo Coelho

**Affiliations:** 1 Internal Medicine, Unidade Local de Saúde de Gaia e Espinho, Vila Nova de Gaia, PRT

**Keywords:** ammonia, hepatic encephalopathy, portal vein arterialization, portosystemic shunt, type b hepatic encephalopathy

## Abstract

Hepatic encephalopathy (HE) is a complex neurological disorder characterized by a wide range of neuropsychiatric abnormalities, from subtle cognitive deficits to deep coma. While cirrhosis is the most common underlying condition, HE can also occur in other conditions. The pathogenesis of HE is multifactorial, but a key factor is the liver's impaired capacity to detoxify substances absorbed from the gut, notably ammonia. The resulting accumulation of these neurotoxins in the systemic circulation leads to their access to the central nervous system, where they disrupt neuronal function.

We present a case report of a 62-year-old woman, with no cognitive impairment, with a relevant medical history of cholangiocarcinoma, surgically treated, with no signs of progression. She was admitted to the Emergency Department (ED) due to confusion and repetitive speech. On neurological examination, the patient presented prostration, with spontaneous eye opening but no directed gaze. She could not name objects upon request and could not repeat or follow commands. She had no cranial nerve deficits and no motor deficits in the limbs. Routine labs including liver function tests were normal except for high ammonia. There was no evidence of infection. A review of her medical history revealed a portal "arterialization" through an anastomosis between the hepatic artery and the portal vein. Subsequently, the patient was diagnosed with HE type B.

This case highlights the importance of a thorough (meticulous) medical history, physical examination, and the patient’s personal background, particularly in patients presenting with acute neurological changes and complex medical histories.

## Introduction

Hepatic encephalopathy (HE) results from liver failure and/or portosystemic shunting and manifests with neurological and psychiatric abnormalities ranging from subclinical symptoms to coma [1]. While cirrhosis is the most common underlying condition, HE can also occur in other conditions. The pathogenesis of HE is multifactorial, but a key factor is the liver's impaired capacity to detoxify substances absorbed from the gut, notably ammonia. The resulting accumulation of these neurotoxins in the systemic circulation leads to their access to the central nervous system, where they disrupt neuronal function.

## Case presentation

A 62-year-old woman, with no cognitive impairment, was admitted to the Emergency Department due to confusion and repetitive speech (palilalia). There was no evidence of focal illness, fever, or other neurological deficits.

The patient's only significant past medical history was cholangiocarcinoma, surgically treated two years prior, with no signs of progression. The patient's only medication was levetiracetam, initiated one month prior for suspected seizures, and supplement use was denied.

In the preceding weeks, the patient presented to the Emergency Department on multiple occasions with similar complaints. Initial evaluations resulted in a diagnosis of urinary tract infection, supported by urine analysis. On other visits, the patient experienced transient aphasia, initially suspected to be seizures. Subsequent treatment with levetiracetam, including dose escalation, for suspected epilepsy did not result in clinical improvement.

On physical examination, the patient was hydrated, afebrile, hemodynamically stable with normal blood pressure and pulse, and well-perfused. Cardiac and pulmonary auscultation was normal, and the abdomen was soft without tenderness on palpation. Peripheral edema was not present. She had no physical evidence of liver disease such as collateral circulation, ascites, or spider angiomas.

On neurological examination, the patient presented prostration, with spontaneous eye opening but no directed gaze. The patient's object naming, repetition, and command following were inconsistent. She had no cranial nerve deficits and no motor deficits in the limbs. Questionable asterixis was present.

Routine labs, including liver function tests, were normal except for high ammonia of 146 μmol/L (reference range: 15-45 μmol/L) (Table 1). Serologies for hepatitis B surface antigen (HBsAg) and hepatitis C antibody (anti-HCV) were negative. Urinalysis was also normal.

**Table 1:** Laboratory results on admission

Laboratory parameter	Result	Reference range
Hemoglobin	12.9 g/dL	12 – 16 g/dL
Leukocyte count	4.4 x10 ^3 ^/uL	3.8 – 10.6 x10 ^3 ^/uL
Platelet count	226 x10 ^3 ^/uL	150 – 440 x10 ^3 ^/uL
Creatinine	0.8 mg/dL	0.67 – 1.17mg/dL
Urea	37 mg/dL	17 – 50 mg/dL
Sodium	138 mmol/L	135 – 145 mmol/L
Potassium	4.1mmol/L	3.5 – 5.5 mmol/L
C-reactive protein	0.38 mg/dL	< 0.5 mg/dL
Alanine aminotransferase	18 U/L	25 U/L
Aspartate aminotransferase	21 U/L	0 – 35 U/L
Alkaline phosphatase	76 U/L	40 – 116 U/L
Gamma-glutamyl transferase	31 U/L	40 U/L
Total bilirubin	0.7 mg/dL	0.3 – 1.0 mg/dL
Prothrombin time	12.3 seg	11.5 - 14.5 seg
Albumin	4.1 g/dL	3.4 – 5.4 g/dL
Ammonia	146 μmol/L	15-45 μmol/L
Anti-Hepatitis C antibody	Negative	
Hepatitis B surface antigen	Negative	

The cranioencephalic computed tomography (CT) scan was unremarkable. Abdominal ultrasound showed no abnormalities and no signs of liver disease. Also, she had a normal upper endoscopy six months prior.

The patient was admitted to the Internal Medicine ward for further evaluation. A lumbar puncture was performed to investigate potential causes, and the cerebrospinal fluid analysis was normal. The review of her medical history revealed that during the resection of cholangiocarcinoma, a complete laceration of the hepatic artery occurred. This was managed with portal "arterialization" through an anastomosis between the hepatic artery and the portal vein. Subsequently, the patient was diagnosed with HE type B (related to portosystemic shunting). The patient responded favorably to treatment with lactulose, supporting the diagnosis.

Following discharge, the patient remained free of HE recurrence and neurological changes for over a year, and she was able to regain her previous level of functional autonomy.

## Discussion

HE can manifest with a broad range of neuropsychiatric abnormalities of varying severity, acuity, and time course with significant clinical implications [2].

HE can be classified based on etiology, severity, and time course. Etiologically, HE is categorized into three types: Type A, associated with acute liver failure; Type B, secondary to portosystemic shunting; and Type C, occurring in the context of cirrhosis. This clinical report represents Type B HE. This portosystemic shunt resulted from a salvage procedure in a previous resection of cholangiocarcinoma. Portal vein arterialization has recently been used as a rescue procedure in hepatopancreatobiliary (HPB) surgery when hepatic artery reconstruction was not possible for oncologic reasons or because of technical limitations [3].

Ammonia has been identified as the principal culprit in HE [4]. However, it is not diagnostic for HE on its own. Its value lies in its high negative predictive value, allowing the exclusion of a diagnosis of HE but not confirming it. When HE is suspected, especially among individuals with chronic liver disease (although this was not the case), a thorough investigation of potential precipitating factors and other possible clinical causes is warranted, given that HE can be associated with precipitating factors and is a diagnosis of exclusion.

## Conclusions

This case underscores the importance of a thorough medical history and physical examination and detailed patient background, particularly in individuals presenting with acute neurological changes and complex medical histories. Furthermore, it highlights that hepatic encephalopathy (HE) is not exclusive to patients with liver disease. Clinicians must be aware of the possibility of a portosystemic shunt in patients with a history of hepatobiliary-pancreatic surgery. Following the diagnosis of HE Type B, treatment was relatively straightforward, resulting in a full response.
